# Progress in the Use of Hydrogels for Antioxidant Delivery in Skin Wounds

**DOI:** 10.3390/pharmaceutics16040524

**Published:** 2024-04-10

**Authors:** Lidia Maeso, Pablo Edmundo Antezana, Ailen Gala Hvozda Arana, Pablo Andrés Evelson, Gorka Orive, Martín Federico Desimone

**Affiliations:** 1 NanoBioCel Research Group, School of Pharmacy, University of the Basque Country (UPV/EHU), 01006 Vitoria-Gasteiz, Spain; lidia.maeso@ehu.eus (L.M.); gorka.orive@ehu.es (G.O.); 2 Consejo Nacional de Investigaciones Científicas y Técnicas, Instituto de Bioquímica y Medicina Molecular (IBIMOL), Universidad de Buenos Aires, Buenos Aires 1113, Argentina; pablo.e.antezana@gmail.com (P.E.A.); agala@docente.ffyb.uba.ar (A.G.H.A.); pevelson@ffyb.uba.ar (P.A.E.); 3 Universidad de Buenos Aires, Facultad de Farmacia y Bioquímica, Departamento de Ciencias Químicas, Cátedra de Química Analítica Instrumental, Buenos Aires 1113, Argentina; 4 Universidad de Buenos Aires, Facultad de Farmacia y Bioquímica, Departamento de Ciencias Químicas, Cátedra de Química General e Inorgánica, Buenos Aires 1113, Argentina; 5 NanoBioCel Research Group, Bioaraba, 01009 Vitoria-Gasteiz, Spain; 6 Biomedical Research Networking Centre in Bioengineering, Biomaterials and Nanomedicine (CIBER-BBN), 01006 Vitoria-Gasteiz, Spain; 7 University Institute for Regenerative Medicine and Oral Implantology—UIRMI (UPV/EHU-Fundación Eduardo Anitua), 01007 Vitoria-Gasteiz, Spain; 8 Consejo Nacional de Investigaciones Científicas y Técnicas, Instituto de Química y Metabolismo del Fármaco (IQUIMEFA), Universidad de Buenos Aires, Buenos Aires 1113, Argentina

**Keywords:** biomaterials, antioxidants, wound healing, regenerative medicine, tissue engineering

## Abstract

The skin is the largest organ of the body, and it acts as a protective barrier against external factors. Chronic wounds affect millions of people worldwide and are associated with significant morbidity and reduced quality of life. One of the main factors involved in delayed wound healing is oxidative injury, which is triggered by the overproduction of reactive oxygen species. Oxidative stress has been implicated in the pathogenesis of chronic wounds, where it is known to impair wound healing by causing damage to cellular components, delaying the inflammatory phase of healing, and inhibiting the formation of new blood vessels. Thereby, the treatment of chronic wounds requires a multidisciplinary approach that addresses the underlying causes of the wound, provides optimal wound care, and promotes wound healing. Among the promising approaches to taking care of chronic wounds, antioxidants are gaining interest since they offer multiple benefits related to skin health. Therefore, in this review, we will highlight the latest advances in the use of natural polymers with antioxidants to generate tissue regeneration microenvironments for skin wound healing.

## 1. Introduction

- Skin and chronic wounds

The skin is the largest organ of the body, covering approximately 16% of body mass [1], and it acts as a protective barrier against external factors such as bacteria, viruses, and physical injuries [2]. Histologically, the skin has two main layers: the epidermis and the dermis. A subcutaneous fascia layer known as the hypodermis resides deep within the dermis. The epidermis is a stratified epithelium that consists of four to five layers of cells, mainly composed of keratinocytes, as well as three other less abundant cell types [3].

**Figure 1:**
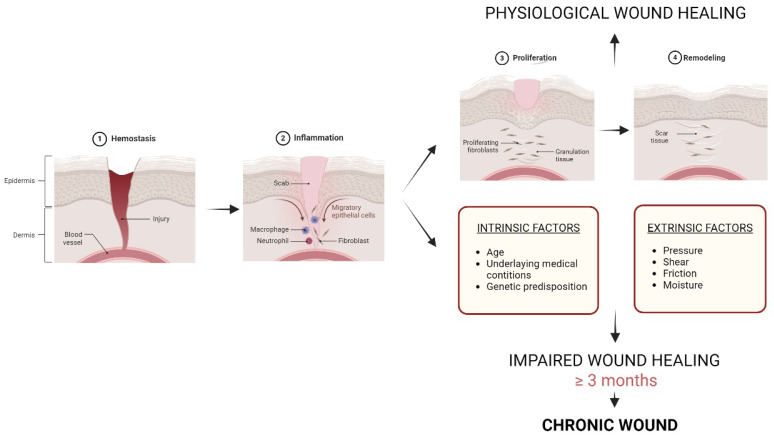
Illustrative image of the phases of wound healing, differentiating between the physiological healing process and the impaired process that leads to the formation of chronic wounds.

When the skin is damaged, the body initiates a complex healing process that involves different cells and molecules. Wound healing is a multifaceted sequential process governed by four overlapping phases, including: hemostasis, inflammation, proliferation, and remodeling (Figure 1) [4]. However, in some cases, the healing process may be impaired, leading to the formation of chronic wounds. Chronic wounds are defined as wounds that fail to heal within 3 months or more, and they affect millions of people worldwide [2,5]. Chronic wounds can be classified into different types based on their etiology and location. The most common types of chronic wounds include pressure ulcers, diabetic foot ulcers, venous ulcers, and arterial ulcers, with the last two typically found on the lower legs [2].

Chronic wounds are associated with significant morbidity and reduced quality of life, and they also impose a significant burden on healthcare systems [6]. According to various research reports and data published by the Mission Regional Medical Center in 2020, it was estimated that around 6.7 million people in the United States were suffering from chronic wounds [7]. Focusing on Europe, it is remarkable that in the United Kingdom, there are 2.2 million patients with wounds, and 25–50% of hospital beds in that country are occupied by patients with wounds. This represents an expense of millions of dollars, which in Europe can account for between 2% and 4% of health outlay [8]. Moreover, in developed countries, it has been estimated that 1 to 2% of the population will experience a chronic wound during their lifetime [9]. In this sense, global chronic wound care market size was valued at USD 11.61 billion in 2021. The market is projected to grow from USD 12.36 billion in 2022 to USD 19.52 billion by 2029, exhibiting a CAGR of 6.7% during the forecast period [10]. Moreover, chronic wounds not only carry a heavy financial burden but also diminish the health-related quality of life of patients. Therefore, there is an urgent need for the development and implementation of wound management strategies that focus on improving health-related quality of life while effectively reducing the costs associated with wound care.

Chronic wounds are caused by a complex interplay between intrinsic and extrinsic factors. Intrinsic factors include age, underlying medical conditions, and genetic predisposition, while extrinsic factors include pressure, shear, friction, and moisture. In addition, chronic wounds are linked to a dysregulated inflammatory response, which translates into long-term inflammation and impaired healing. A prolonged inflammatory response is characterized by the accumulation of pro-inflammatory cytokines, such as tumor necrosis factor-alpha (TNF-α), interleukin-1 beta (IL-1β), and the damaged expression of growth factors, e.g., transforming growth factor-beta (TGF-β) and vascular endothelial growth factor (VEGF). Another characteristic of chronic wounds includes the dysregulation of the extracellular matrix (ECM), which provides structural support to the skin; in particular, the expression of matrix metalloproteinases (MMPs) is increased, leading to a major degradation of ECM proteins (mainly collagen) at the same time that the expression of tissue inhibitors of MMPs (TIMPs) is decreased, affecting the ECM synthesis and degradation balance and leading to a net loss of ECM [11,12].

b. Oxidative stress and its role in chronic wounds

One of the main factors involved in delayed wound healing is oxidative injury, which is triggered by the overproduction of reactive oxygen species (ROS). This is even more important in this process, as it involves several factors, such as fibroblasts, endothelial cells, blood cells, continuous inflammatory processes, and granulation and tissue remodeling, in which ROS can cause noteworthy dysregulation [13]. Oxidative stress can be defined as an imbalance between oxidants and antioxidants in favor of the oxidants, leading to a disruption of metabolic signaling and transcription processes, which results in oxidative damage to macromolecules [14]. Most common ROS include superoxide anion (O_2_^●−^), peroxides (hydrogen peroxide and organic peroxides), hydroxyl radical (OH^●^), and singlet oxygen (^1^O_2_), an excited state of molecular oxygen [15]. Oxidative stress has been implicated in the pathogenesis of chronic wounds, where it is known to impair wound healing by causing damage to cellular components, delaying the inflammatory phase of healing, and inhibiting the formation of new blood vessels [16].

Many studies have shown that low levels of ROS are conducive to normal wound healing by stimulating cell migration and angiogenesis, but excessive ROS can hinder or even endanger wound healing, especially in chronic wounds [11,12,17,18,19]. Long-term instability and high concentrations of ROS will eventually lead to angiogenesis pathological damage, making blood supply and nutritional requirements unable to meet the needs of wound healing [17,20]. An additional noteworthy point is that ROS, as a crucial regulator of the wound healing process, is required at low levels to mediate intracellular signaling. However, excessive amounts are produced in wounded and inflamed tissue by NADPH oxidase, an enzyme complex, which is expressed at particular high levels by inflammatory cells [21]. Moreover, oxidative stress, being a consequence of free radicals’ increments, could lead to neutrophil recruitment and the secretion of proinflammatory cytokines. In this vein, antioxidant molecules play an important role in protecting the cell from reactive oxidant species and thereby promoting wound healing [22]. 

Within this framework, hydrogels are proposed as an ideal candidate for wound healing due to their 3D structure, good permeability, excellent biocompatibility, and their ability to provide a moist environment for wound repair [23,24]. In this sense, during the wound healing process, hydrogels should protect the injury and support tissue recovery. Advances in the areas of technology and biomaterials have facilitated the use of engineered constructs for medical issues, such as biopolymers that can easily form hydrogels and mimic the molecules that comprise the extracellular matrix, widely used in the fields of tissue engineering and regenerative medicine. In this vein, many antioxidant-releasing hydrogels have emerged [25].

Thereby, the treatment of chronic wounds requires a multidisciplinary approach that addresses the underlying causes of the wound, provides optimal wound care, and promotes wound healing. Advanced wound care products and emerging therapies, such as growth factors, antioxidants, and stem cells, show promise in promoting wound healing and improving the outcomes of patients with chronic wounds. Therefore, in this review we will highlight the latest advances in the use of natural polymers with antioxidants to generate tissue regeneration microenvironments for skin wound healing.

## 2. Antioxidants, a Skin Ally

Antioxidants can be defined as any substance that delays, prevents, or removes oxidative damage to a target molecule [26]. In this sense, antioxidants play a crucial role in protecting the body against oxidative stress, a process linked to various diseases, such as chronic wounds and aging. Antioxidants can be classified into two major groups: antioxidants enzymes, such as superoxide dismutase (SOD), catalase, and glutathione peroxidase (GPx), among others, and non-antioxidants enzymes [14]. In this review, we are going to emphasize the second group and their use in the wound healing process. Non-enzymatic antioxidants exert their effects through different mechanisms. Understanding these mechanisms is crucial for harnessing the full potential of antioxidants for skin health: (a) free radical scavenging (antioxidants donate electrons to neutralize free radicals, preventing them from damaging cellular structures such as DNA, proteins, and lipids), (b) enzyme regulation (certain antioxidants play a role in enzyme function, enabling them to participate in cellular energy production and antioxidant defense), (c) chelation (some antioxidants can bind to metal ions, reducing their ability to catalyze harmful oxidative reactions), and (d) gene expression (antioxidants can modulate gene expression, influencing the production of endogenous antioxidants and other protective proteins) [26,27,28,29]. Table 1 shows some of the main skin health-related non-enzymatic antioxidants, describing their main antioxidant mechanisms, as well as their solubility and sources. 

The role of oxidative stress in various skin diseases is becoming increasingly apparent, and there is growing evidence to support the effectiveness of antioxidative strategies in the management of these conditions. Such strategies offer a simple and effective means of improving skin health [30]. 

Furthermore, it is well known that antioxidants offer multiple benefits relating to skin health other than wound healing, such as: (a) anti-aging properties (antioxidants combat the breakdown of collagen and elastin, key proteins responsible for skin elasticity and firmness) [31], (b) sun protection (certain antioxidants, like vitamin C and E, provide mild protection against UV-induced damage, supplementing the effects of sunscreen) [27,28], (c) skin brightening (antioxidants can inhibit the activity of tyrosinase, an enzyme involved in melanin production, leading to a more even skin tone and reduced hyperpigmentation) [28], (d) anti-inflammatory effects (many antioxidants possess anti-inflammatory properties, soothing irritated skin and reducing redness and swelling) [28,31], and (e) protection against environmental stressors (by neutralizing free radicals generated by environmental pollutants and toxins, antioxidants shield the skin from environmental stressors) [27,28].

Within this framework, several studies have investigated the use of antioxidants for wound healing. Curcumin, N-acetylcysteine (NAC), and quercetin are some of the antioxidant compounds that have shown initial evidence of efficacy. Curcumin, a polyphenol found in turmeric, has been shown to promote wound healing by reducing inflammation and oxidative stress. NAC, a precursor of glutathione, can increase the levels of glutathione in the body and reduce oxidative stress. Quercetin, a flavonoid found in fruits and vegetables, can scavenge free radicals and inhibit inflammation [32,33].

Antioxidants can be broadly categorized into hydrophilic, hydrophobic, and amphiphilic (when the molecule has both hydrophobic and hydrophilic regions) compounds based on their solubility properties. On one hand, antioxidants that are water-soluble react with oxidants in the cell cytoplasm as well as in the blood plasma. On the other hand, lipid-soluble antioxidants are responsible for protecting cell membranes from lipid peroxidation [34]. Nevertheless, both have a great capacity for regulating the redox state by restraining and/or retarding the oxidation of other substrates, although the effectiveness of each antioxidant may vary based on its stability, bioavailability, and interaction with other compounds [35]. An additional noteworthy point is that antioxidants often exhibit synergistic effects, reinforcing each other’s antioxidant activities. For instance, hydrophilic antioxidants, such as vitamin C and glutathione, can regenerate oxidized hydrophobic antioxidants like vitamin E, ensuring their sustained antioxidant function [36,37,38,39].

- Hydrophilic antioxidants

Hydrophilic antioxidants are water-soluble compounds that readily dissolve in the aqueous environment of biological systems. Thus, they are mainly found in the cytoplasm and extracellular fluid. Notable hydrophilic antioxidants include ascorbic acid (vitamin C), melatonin, and glutathione [34].

a.1. Ascorbic acid

Ascorbic acid, better known as vitamin C, is a powerful antioxidant that can help protect the wound environment from oxidative damage. This noteworthy antioxidant capacity comes from its ability to neutralize free radicals [27,40], as well as from the fact that ascorbic acid regenerates other antioxidants like vitamin E, further enhancing the overall antioxidant capacity of cells [40,41].

Ascorbic acid is essential for the synthesis of collagen, a structural protein in the skin, which is fundamental for tissue repair [42,43]. Studies demonstrate that vitamin C promotes wound healing via different mechanisms, among which the recently mentioned collagen synthesis stands out, as well as cell migration and transformation, with antioxidant activity being rapidly consumed post-wounding [41,42].

Additionally, ascorbic acid can modulate the inflammatory response of the body, and it is well known that excessive inflammation can delay the wound healing process, although inflammation is a critical phase of the body’s response to injury and infection. In this vein, the effects of vitamin C on inflammation and wound healing have been studied in a mice model where vitamin C reduced inflammation by favorably modulating macrophage function, improving wound healing outcomes [44]. Additionally, vitamin C supports the immune system, which is vital for fighting off infections that could impair wound healing. In this sense, a recent review article explored the beneficial impact of vitamin C on immune function and wound healing [45]. 

Regarding clinical trials, Sarpooshi and collaborators showed vitamin C’s significant beneficial impact on improving wound healing in second-degree burn-referred patients. 

**Table 1:** Solubility, classification, mechanism, and sources of skin health-related main non-enzymatic antioxidants.

Antioxidant	Solubility	Classification	Mechanism	Source	Refs.
Ascorbic acid (Vitamin C)	Water-soluble	Endogenous and exogenous	Free radical scavenger, regenerates vitamin E	Fruits and vegetables, especially citrus fruits, strawberries, kiwi, mango, papaya, pineapple, bell peppers, broccoli, Brussels sprouts, and tomatoes	[46,47,48,49]
Melatonin	Water-soluble	Endogenous	Free radical scavenger, induces the activity of some antioxidant enzymes	Produced endogenously in the body, dietary sources include eggs, fish, nuts, milk, grapes, mushrooms, oats, corn, tart cherries, and tomatoes	[46,50]
Glutathione (GSH)	Water-soluble	Endogenous	Free radical scavenger, regenerates other antioxidants, detoxification	Produced endogenously in the body, dietary sources include fruits and vegetables, especially avocados, asparagus, spinach, and okra	[46,51]
Alpha-Tocopherol (Vitamin E)	Lipid-soluble	Exogenous	Free radical scavenger, regenerates vitamin C	Found in nuts, seeds, vegetable oils, and leafy green vegetables	[46,52]
Ubiquinone (Coenzyme Q10)	Lipid-soluble	Endogenous and exogenous	Free radical scavenger, protects cell membranes	Found in small amounts in meats, fish, and whole grains, and also available as a dietary supplement	[46,53]
Carotenoids	Lipid-soluble	Exogenous	Free radical scavenger, singlet oxygen quenching, protection against exposure to UV radiation, regenerates vitamin E and vitamin C	Found in fruits and vegetables, especially those that are red, orange, and yellow in color, and also available as a dietary supplement	[46,54]
alpha-lipoic acid (ALA)	Water and lipid-soluble	Endogenous and exogenous	Free radical scavenger, regenerates other antioxidants, metal chelation, effect on gene expression	Found in small amounts in foods such as spinach, broccoli, and potatoes, and also available as a dietary supplement	[46,55]
Flavonoids and other polyphenols	Water and lipid-soluble	Exogenous	Peroxyl radical scavenger, metal chelation	Found in fruits, vegetables, tea, wine, and chocolate	[56]

a.2. Melatonin

Melatonin is a hormone produced naturally by the pineal gland in the brain, whose main function is to regulate sleep–wake cycles, that is, circadian rhythms. However, it also possesses antioxidant properties, mainly because it is a potent free radical scavenger. This can be beneficial for health processes, including wound healing [50,57].

Melatonin’s antioxidant capacity is attributed to several mechanisms:

- Direct free radical scavenging: This is the main mechanism by which melatonin can directly neutralize a variety of free radicals and reactive oxygen species, reducing their harmful effects on cells and tissues [50,58].
- Indirect antioxidant effects: Melatonin also stimulates the activity of antioxidant enzymes, such as superoxide dismutase (SOD) and glutathione peroxidase (GPx), which further enhance the body’s antioxidant defense systems [50].
- Mitochondrial protection: Melatonin can protect mitochondria from oxidative damage, helping maintain cellular energy production, which is essential for healing processes [59,60,61,62,63].

A study conducted by Pugazhenthi et al. investigated the effects of melatonin on wound healing in rats. The study found that melatonin supplementation accelerated wound closure and improved tissue regeneration. It also noted reduced oxidative stress and increased antioxidant enzyme activity in the melatonin-treated group [64]. A follow-up study by Song and collaborators explored the role of melatonin in diabetic wound healing in vitro by using high glucose (HG)-cultured keratinocytes, showing a pro-proliferative, pro-migratory, and anti-apoptotic effect of melatonin on HG-treated keratinocytes, which was mediated by the extracellular signal-regulated kinase signaling pathway [65]. 

a.3. Glutathione

Glutathione is a tripeptide composed of three amino acids: glutamine, cysteine, and glycine. It is found in every cell in the body and is well known for its ability to neutralize free radicals and reactive oxygen species. Its antioxidant capacity is critical for maintaining cellular health and has been linked to various physiological processes, including wound healing. In fact, deficiencies in glutathione have been associated with increased susceptibility to oxidative stress-related diseases, such as neurodegenerative disorders and liver damage [66].

Glutathione functions as an antioxidant through several mechanisms:

- Direct free radical scavenging: Glutathione can directly interact with free radicals and neutralize them, such as superoxide radicals and hydroxyl radicals, by donating electrons [51,67,68].
- Regeneration of other antioxidants: Glutathione is involved in the regeneration of other antioxidants, such as vitamin C and E. It can recycle these antioxidants, making them available for further free radical neutralization [51,67,68].
- Detoxification: Glutathione plays a crucial role in the detoxification of harmful substances and metabolic products. Indeed, it is attached to toxins by the enzyme glutathione s-transferase (GST), thus facilitating their removal from the body [51,67].

A study published by Tamer et al. aimed to formulate a wound dressing polyelectrolyte membrane based on chitosan (Ch) and sodium hyaluronate (HA) loaded with glutathione (GSH). The wound healing process that was examined using a standard rat model exhibited progress when using these membranes compared to the ones without glutathione [69].

b. Hydrophobic antioxidants

Hydrophobic antioxidants, unlike the previously described hydrophilic ones, are lipid-soluble and consequently, are found primarily in cell membranes and lipoproteins. They protect the lipidic components of cells from oxidative damage. Notable hydrophobic antioxidants include alpha-tocopherol (vitamin E), ubiquinone (coenzyme Q10), and carotenoids, among others [34].

b.1. Alpha-tocopherol

Alpha-tocopherol, commonly known as vitamin E, is a fat-soluble antioxidant that plays a crucial role in protecting cells and tissues from oxidative damage. It is the most biologically active form of vitamin E and is found in different foods, especially nuts, seeds, and vegetable oils. It acts primarily as a lipid peroxyl radical scavenger, preventing the propagation of lipid oxidation chain reactions [52]. Alpha-tocopherol can directly neutralize free radicals, such as peroxyl radicals and lipid peroxides, by donating electrons. This helps prevent oxidative damage to cell membranes, lipids, and proteins. Alpha-tocopherol works synergistically with other antioxidants, such as vitamin C, to regenerate their antioxidant forms, further enhancing the body’s overall antioxidant defense system [29,70,71].

A study conducted by Chong and collaborators investigated the effects of a tocotrienol-based nanoemulsified (NE) system topical vitamin E (alpha-tocopherol) on zebrafish tail regeneration. The study reported that after 24 h of treatment, the wound closure of keratinocytes was found to be significantly faster by 73.76%, 63.37%, and 35.56%, respectively, when treated with 3.50 μg/mL and 1.75 μg/mL of NE compared to the blank, showing the potential of the tocotrienols (as alpha-Tocopherol) system in enhancing wound healing through accelerated wound closure [72].

b.2. Ubiquinone

Ubiquinone, also known as coenzyme Q10 (CoQ10), is a lipid-soluble compound mostly found in the mitochondria of cells. It plays a critical role in the electron transport chain, which is part of the process of cellular respiration and energy production. This helps maintain efficient energy production and cell function. Additionally, ubiquinone has antioxidant properties, primarily related to its role in protecting cells from oxidative damage. Moreover, ubiquinone can recycle alpha-tocopherol, providing antioxidant protection to membranes [73,74,75,76,77,78].

Some studies have examined the effect of Q10 topical treatment, showing that this compound is able to penetrate skin and can significantly reduce the elevated levels of free radicals, indicating an antioxidant effect of topical Q10 application, as well as an ability to support the maintenance of cellular energy levels [53]. 

b.3. Carotenoids

Carotenoids, including beta-carotene, lutein, lycopene, and zeaxanthin, serve as antioxidants in the body through several mechanisms: free radical scavenging, singlet oxygen quenching, protection against photooxidation caused by exposure to ultraviolet (UV) radiation, and enhancement of antioxidant defenses, including vitamin E and vitamin C, by regenerating their active forms [54,79,80,81].

Particularly, lycopene is a naturally occurring red carotenoid pigment found in various fruits and vegetables, with tomatoes and tomato-based products being particularly rich sources. Lycopene’s antioxidant capacity stems from its ability to neutralize free radicals and reactive oxygen species. Lycopene has a particular affinity for lipid-rich cell membranes, where it helps protect polyunsaturated fatty acids from lipid peroxidation, a key mechanism of oxidative damage [82,83,84]. There are several studies that prove the antioxidant activity of lycopene, showing its effects on diverse molecular pathways related with oxidative stress [85,86,87,88,89].

On the other hand, in a clinical study conducted by Palombo et al., the administration of lutein and zeaxanthin, either orally, topically, or in combination, showed a significant improvement in the prevention of skin lipid peroxidation and photoprotective activity related to free radicals compared to the placebo group [90].

c. Amphiphilic antioxidants

c.1. Alpha–lipoic acid

Alpha–lipoic acid (ALA) is a compound that functions as a powerful antioxidant in the body. It is synthesized in mitochondria, and it can also be absorbed from the gut. ALA is unique among antioxidants because it is both water-soluble and lipo-soluble, which allows it to work in various cellular environments [55].

ALA’s antioxidant capacity is attributed to several mechanisms [55,91,92]:

- Free radical scavenging: ALA can directly neutralize a wide range of free radicals and reactive oxygen species by donating electrons. This is helpful when preventing oxidative damage to lipids, proteins, and also DNA.
- Regeneration of other antioxidants: ALA is able to regenerate and enhance the activity of other antioxidants, such as vitamin C and E, glutathione, and coenzyme Q10.
- Metal chelation: ALA can bind to certain metal ions, such as iron and copper. In this manner, it reduces their ability to promote the formation of harmful ROS.
- Effects on gene expression: ALA can induce the expression of antioxidant enzymes through nuclear factor E2-related factor 2 (Nrf2).

A systematic review and meta-analysis investigated the antioxidant effects of alpha-lipoic acid and its efficacy in reducing ROS. The study found that ALA, indeed, has an antioxidant effect and can reduce oxidative stress parameters [93].

c.2. Flavonoids and other polyphenols

Flavonoids and other polyphenols are derived from plants. The antioxidant activity of phenols relies on their capacity to inhibit lipid peroxidation by acting as peroxyl radical scavengers that stop the chain reaction. In addition, some phenols have the ability to chelate or bind to metal ions, such as iron and copper. This helps prevent these metals from catalyzing the formation of ROS [26].

In this context, flavonoids are a diverse group of polyphenolic compounds that can be found in various plant-based foods, such as fruits, vegetables, tea, and red wine. Furthermore, some other flavonoids can also stimulate the activity of endogenous antioxidant enzymes, such as superoxide dismutase and glutathione peroxidase [56,94,95,96,97,98]. 

Epigallocatechin gallate (EGCG) is a flavonoid that is abundant in green tea. It has been widely studied for its antioxidant properties. In fact, some studies have reported the wound healing potential of EGCG through different mechanisms, including its ability to inhibit macrophage-mediated inflammation via the Notch signaling pathway [99], enhance wound healing in diabetic mice by accelerating re-epithelialization and angiogenesis [100], and improve wound healing in diabetes [101]. A novel wound dressing based on EGCG self-assembled hydrogels has also been developed to promote wound healing [102]. Thus, EGCG has been proven to be a potent antioxidant effective for wound healing purposes [103].

Quercetin is another well-known antioxidant flavonoid found in various fruits and vegetables. A research study by Gopalakrishnan et al. examined the effects of quercetin on wound healing in rats and reported that quercetin supplementation improved wound closure, increasing levels of VEGF and TGF-β1, thereby effectively modulating the cytokines, growth factors, and cells involved in some of the phases of healing [104]. 

Resveratrol is a natural polyphenolic compound that can be found most notably in grapes, red wine, and berries. Its antioxidant ability is due, on one hand, to its capacity to directly neutralize free radicals, such as superoxide anions and hydroxyl radicals, by donating electrons. This reduces oxidative damage to cellular components. On the other hand, resveratrol also increases the activity of antioxidant enzymes, such as superoxide dismutase and catalase, which play crucial roles in neutralizing reactive oxygen species [105]. Yoksa and collaborators investigated the use of 5% topical resveratrol ointment in the management of burn wounds of adult albino rats. The study reported that resveratrol promoted wound closure through collagen fiber synthesis, granulation tissue formation, and epithelial regeneration [106].

Curcumin is a natural polyphenolic compound found in the rhizomes of turmeric (*Curcuma longa*). Its antioxidant properties, which stem from its capacity to neutralize free radicals and reactive oxygen species in the body, as well as from its anti-inflammatory properties, are remarkable and have been extensively studied for their potential benefits in human health. Moreover, it contributes to the production and activity of antioxidant enzymes and their constituents, such as glutathione [107,108]. Multiple studies analyzed the potential effect of curcumin on wound healing in animal models, showing that curcumin accelerates wound healing by reducing inflammation, increasing collagen deposition, and promoting angiogenesis [109,110,111]. As an example, Kant et al. studied the effect of curcumin-loaded gel topically applied on diabetic wounded rats, demonstrating that curcumin application increased wound contraction and decreased the expression of inflammatory cytokines and enzymes and increased the levels of the anti-inflammatory cytokine IL-10 and antioxidant enzymes (i.e., superoxide dismutase, catalase, and glutathione peroxidase). The results of this study indicate that the anti-inflammatory and antioxidant properties of curcumin contributed to improved wound healing in diabetic rats, resulting in faster and more effective healing [112]. 

Considering the marshaled information, it is clear that non-enzymatic antioxidants play critical roles in maintaining cellular redox balance and protecting against oxidative stress-related damage. Thereby, these antioxidants can be an effective strategy for treating chronic wounds when combined with hydrogel patches that allow for their sustained and efficient release. 

## 3. Hydrogels for Chronic Wound Repair

Hydrogels are essentially 3D networks that are intensely hydrated with solid-like properties, and they are primarily comprised of crosslinked hydrophilic polymers [113]. In addition, hydrogels can absorb water excess due to their hydrophilic porous structure. Moreover, they display similarities to the native extracellular matrix [114,115]. In this sense, due to their versatile characteristics, hydrogels have emerged as highly suitable scaffolds with great promise in the field of biomedicine. Their appeal lies in their capacity for customization and, notably, their exceptional biocompatibility and biodegradability [116,117]. Hydrogels can be derived from both natural sources, including collagen, alginate, hyaluronic acid, chitosan, and gelatin, as well as synthetic materials like poly(ethylene glycol), poly(vinyl alcohol), and poly(lactic acid) [25,118,119] (summarized in Table 2).

- Natural polymer hydrogels

A wide range of different natural polysaccharides and proteins can be used in the development of hydrogels, since theu are abundant in nature [120,121]. Natural polymers are highly regarded in the field of skin tissue engineering, primarily due to their excellent biocompatibility and degradability [122]. However, it is important to note that natural polymers often exhibit suboptimal mechanical properties. In order to address this limitation, modifications such as grafting, multiple crosslinking, and blending with other polymers are commonly employed to enhance their mechanical characteristics [123].

a.1. Collagen

Collagen has garnered significant attention due to its remarkable properties. It is the most abundant protein found in the extracellular matrix of animals, constituting approximately 30% of the protein content in vertebrates. Collagen plays a key role in maintaining the biological and structural integrity of the extracellular matrix [124,125]. This protein serves a dual function, combining structural and functional roles. In this vein, it imparts strength, flexibility, and stability to tissues within the body, ensuring their integrity [126]. Collagen Type I, among other natural polymers, is particularly notable for its exceptional biodegradability, low antigenicity, and biocompatibility. It has the unique ability to self-assemble and crosslink, forming robust and stable fibers. This characteristic makes it a promising candidate for the development of scaffolds [127,128,129].

In particular, collagen hydrogels have garnered significant attention, since they can absorb substantial amounts of water and hydrophilic active molecules and, in the context of wound healing, this could lead to the absorption of large amounts of exudate, leading to less protein and electrolytes lost, helping prevent wound dehydration [130,131]. In addition, they can be enzymatically degraded by matrix metalloproteinases, such as collagenases. These features are essential for designing advanced biomaterials with applications in wound care and the delivery of therapeutic agents [132]. 

Antezana et al. developed a collagen hydrogel loaded with silver nanoparticles and an extract from *Cannabis sativa* oil. The incorporation of silver nanoparticles enhanced its mechanical properties and resistance to collagenase degradation. Most notably, the presence of silver nanoparticles ensures a long-lasting antimicrobial effect. Furthermore, the addition of *Cannabis sativa* oil extract, known for its anti-inflammatory and analgesic properties, along with antioxidant activity, not only improves biocompatibility but also enhances the antimicrobial activity of the resulting nanocomposite [133].

a.2. Alginate

Alginate, also known as alginic acid salt, is a widely used and abundant biopolymer found in nature [134]. It is extracted from the cell walls of brown seaweed and can also be obtained from the capsules of certain microorganisms like *Azotobacter* sp. and *Pseudomonas* sp. Alginate is a linear anionic polysaccharide known for its excellent gelation properties. It can easily solidify by interacting with divalent ions, all without the need for additional chemical crosslinkers [135,136]. Alginate consists of linear copolymers made up of (1,4)-linked β-D-mannuronic (M) and (1,4)-α-L-guluronic (G) acid units. These units are arranged in various configurations, including M-blocks, G-blocks, and sequences with alternating M and G residues. When the carboxylate groups of G residues from purified alginates crosslink with divalent cations such as Mg^2+^, Ba^2+^, Ca^2+^, and Sr^2+^, hydrogels can be formed. Therefore, alginates with a high G concentration typically yield more rigid hydrogels, while those with a lower G content result in softer, elastic materials [137].

Due to its structural similarity to the extracellular matrix, as well as its biocompatibility, lack of toxicity, biodegradability, cost-effectiveness in extraction, and ease of gelation, alginate-based hydrogels are considered excellent candidates for the design and development of scaffolds [135,138]. However, because of its inherent limitations, such as poor stability and relatively soft mechanical properties, it is a common practice to combine alginate with other polymers to augment its overall properties [139,140]. 

Stubbe et al. developed gelatin–alginate hydrogels for burn wound treatment, where the introduction of alginate into the gelatin matrix leads to phase separation with polysaccharide microdomains in a protein matrix. In vitro tests demonstrate better cell adhesion for films with lower alginate content, which also exhibit superior mechanical properties and good biocompatibility [141].

a.3. Hyaluronic acid (HA)

Hyaluronic acid (HA) is a natural heteropolysaccharide that belongs to the glycosaminoglycans group and is composed of repeating disaccharide units, which include a uronic sugar (β−1,4-D-glucuronic acid) and an amino sugar (β−1,3-N-acetyl glucosamine) [142]. HA is one of the essential components of the extracellular matrix and plays a pivotal role in filling organ spaces, cushioning against impact, and lubricating mobile tissues [143]. Beyond its structural and physiological contributions to connective tissues and bodily fluids, HA is involved in various biological processes, including inflammation, morphogenesis, homeostasis, tissue regeneration, maintenance of extracellular matrix integrity, and acts as a signaling molecule that regulates cell adhesion, migration, and proliferation [144,145].

HA is synthesized by hyaluronan synthases and can have a wide range of molecular weights depending on its source [146]. HA exists in the form of a negatively charged hyaluronate macromolecule under normal physiological conditions. This polyanionic hyaluronan is extremely hydrophilic, with the capacity to interact with water significantly more than neutral polymers, enhancing its compatibility with various intra- and extracellular tissue components [147].

HA serves as an ideal biomaterial for the design and development of non-adhesive, non-thrombogenic, and non-immunogenic scaffolds, particularly for applications in tissue engineering and wound dressing, since it presents advantageous properties, such as biocompatibility, biodegradability, bioresorbability, high viscosity, and mechanical stability [148,149].

Zhao et al., developed a photo-responsive supramolecular polysaccharide hydrogel through host–guest interactions between azobenzene and β-cyclodextrin groups linked to hyaluronic acid chains. When exposed to ultraviolet light, the hydrogel loosened, enabling the release of epidermal growth factor, thereby improving EGF delivery at the wound site. This was probed using an in vivo model, where the controlled release of EGF from the supramolecular hydrogel demonstrated superior efficiency in wound healing, as evidenced by enhanced granulation tissue formation, growth factor levels, and angiogenesis. Consequently, these proposed hydrogels hold potential as controlled delivery systems for future clinical applications in wound healing [150].

a.4. Chitosan

Chitosan, a biopolymer derived from the deacetylation of chitin, is a polysaccharide comprising randomly distributed monomeric units of β-(1–4)-D-glucosamine and N-acetyl-D-glucosamine. This material can be used in tissue engineering applications due to its exceptional biodegradability and biocompatibility [151,152,153]. Among the attributes of chitosan, it presents non-toxicity, non-antigenicity, inertness, bioadhesiveness, and hemostatic effects [154]. Furthermore, it can be modified to create multifunctional constructs that closely resemble the natural matrix [155,156]. Notable examples of such derivatives include N-carboxymethyl, N-succinyl, N-carboxybutyl, N-acyl, N-carboxyethyl, N-N-dicarboxymethyl, O-succinyl, and O-carboxymethyl chitosan derivatives, among others [157].

Chen et al. studied the impact of SIKVAV-modified chitosan hydrogels on skin wound healing using a mouse model with induced skin wounds. They observed that the application of SIKVAV-modified chitosan hydrogels accelerated wound reepithelialization compared to control groups. In addition, there was an increased deposition of myofibroblasts in wounds treated with SIKVAV-modified chitosan hydrogels. Additionally, these hydrogels demonstrated the promotion of angiogenesis, keratinocyte proliferation, and differentiation, along with the inhibition of inflammation in skin wounds. These findings suggest that modified chitosan hydrogels could be a valuable component in the treatment of wounds [158].

a.5. Gelatin

Gelatin is produced through acidic or basic partial hydrolysis of collagen, a predominant protein found in the natural extracellular matrix [159,160]. Gelatin-based hydrogels have garnered considerable attention and application due to their biocompatibility, biodegradability, degradation mediated by matrix metalloproteinases, preservation of natural cell adhesion motifs, low antigenicity, and minimal inflammatory response when introduced in vivo due to its degradation process [161,162,163,164]. Furthermore, the diverse functional groups present in gelatin, including primary amine, carboxyl, and hydroxyl groups, allow for modifications with various crosslinkers or therapeutic agents, expanding its versatility as a material applicable to wound healing and tissue regeneration. Various crosslinking strategies have been developed for the preparation of in situ gellable gelatin-based hydrogels, involving both physical and chemical crosslinking reactions [165,166,167]. Nevertheless, the complex preparation procedure associated with these hydrogels often limits the full demonstration of their exceptional biological activity. Consequently, there is a significant need to investigate more stable and effective gelatin-based hydrogel dressings [168].

Dong et al. developed an injectable hydrogel composed of poly(ethylene glycol) (PEG) and gelatin, featuring highly adjustable properties derived from a multifunctional PEG-based hyperbranched polymer and commercially available thiolated gelatin. They encapsulated murine adipose-derived stem cells in the hydrogel; this allowed for the cells’ growth while preserving their stemness. By modifying the hydrogel formulation and cell seeding densities, the authors could control mechanical properties, biodegradability, and cellular responses. In addition, the use of the animal model demonstrated that the hydrogel, formed in situ, significantly enhances cell retention, promotes angiogenesis, and accelerates wound closure. These findings indicate that the injectable PEG–gelatin hydrogel has potential applications in regulating stem cell behaviors in 3D culture and delivering cells for wound healing and other tissue regeneration purposes [169].

b. Synthetic polymer hydrogels

Synthetic polymers are polymers that are artificially created through chemical reactions, and they possess adjustable chemical and physical characteristics [170]. In comparison to natural biopolymers, synthetic polymers exhibit superior mechanical properties. They can be readily modified to enhance their physicochemical attributes and can also be functionalized with various molecules to fulfill specific requirements [171].

b.1. Poly(ethylene glycol) (PEG)

PEG is a polymer of ethylene oxide, formed when ethylene oxide reacts with ethylene glycol, ethylene glycol oligomers, or water [172]. In addition, it is a non-ionic and biocompatible material with optimal physicochemical and biological attributes, making it well-suited for biomedical applications. It is particularly well-suited for biological applications, as it typically does not generate an immune response [173]. PEG hydrogels can be prepared through various crosslinking methods [174]. In this sense, these hydrogels can be used for drug delivery and as vehicles for cell delivery to facilitate tissue regeneration [175]. The choice of crosslinking process significantly influences the physicochemical properties of the hydrogels, including permeability, molecular diffusion, elasticity, modulus, or degradation rate. Chemical modification of PEG scaffolds can further enhance their biological properties [176]. 

Jafari et al. developed a hydrogel for the healing process of full-thickness wounds. The hydrogel was made of chitosan and maleic-terminated polyethylene glycol (PEG-MA), where PEG-MA was synthesized by reacting PEG with maleic anhydride. The addition of TiO_2_ nanoparticles to the matrix enhanced the properties of the hydrogel. The hydrogels exhibited a porous structure with a swelling ratio in the range of 240–280%. These hydrogels demonstrated support for human fibroblast cell proliferation over a tested period of 7 days. In addition, in vivo biocompatibility and full-thickness wound closure tests further substantiated the hydrogels’ in vivo biocompatibility and accelerated wound closure in rat models, respectively [177]. 

b.2. Poly(vinyl alcohol) (PVA)

PVA is a synthetic polymer known for its high hydration, water solubility, and the ability to form hydrogels. PVA hydrogels can be created through physical crosslinking using methods like repeated freezing/thawing or through chemical crosslinking with agents such as glutaraldehyde or epichlorohydrin. PVA can also undergo modification using acryloyl chloride or glycidyl methacrylate to introduce reactive acrylate groups. Additionally, PVA has the versatility to blend with other water-soluble polymers, resulting in the creation of composite hydrogels [178]. PVA hydrogels exhibit reduced protein-binding tendencies, relatively higher elasticity, and water content. In the medical field, PVA serves as a biomaterial owing to its biocompatibility, non-carcinogenic properties, non-toxicity, swelling characteristics, and bioadhesive features [179,180,181]. 

Khorasani et al., developed a polyvinyl alcohol/chitosan/nano zinc oxide nanocomposite hydrogel using the freeze–thaw method. In this sense, an increase in freeze–thaw cycles led to reduced pore size and increased porosity and wound fluid absorption. Furthermore, increased freeze–thaw cycles and reduced thawing temperatures resulted in an increase in elastic modulus and tensile strength, while elongation at the break point decreased. Finally, these hydrogels showed antibacterial properties, biocompatibility, non-toxic effects, and effectively contributed to wound treatment [182].

b.3. Poly(lactic acid) (PLA)

PLA and its copolymers, being hydrophobic polyesters, find extensive use in biomedical applications. Given that lactides lack functionality, they are often copolymerized with hydrophilic monomers or conjugated with hydrophilic moieties to create hydrogels. The production of PLA requires the use of pure D-lactide, L-lactide, or their combinations [183,184]. The two stereoisomers of lactic acid (LA) can give rise to four types of polylactic acid (PLA): poly(L-lactic acid) (PLLA), poly(D-lactic acid) (PDLA), poly(D,L-lactic acid) (PDLLA), and meso PLA. PLLA and PDLA exhibit hemi-crystalline and crystalline characteristics, respectively, with a regular chain structure. On the other hand, PDLLA is amorphous [185]. PLA presents high hydrophobicity, which limits its application in the biomedical field. To overcome this limitation and enhance the utility of PLA hydrogels in biomedical applications, modifications to the PLA attributes can be made by copolymerizing or crosslinking with other polymers such as polyethylene oxide, polyethylene glycol, polysaccharides, and polypeptides, among others [186]. These modifications aim to improve the hydrophilicity and overall performance of PLA-based materials in biomedical applications. 

Although an interesting advantage of PLLA is its degradation into non-toxic by-products and its ability to be easily combined with other materials, PLLA exhibits a faster degradation rate and a relatively lower degradation rate compared to other materials used in tissue engineering. The degradation products of PLLA could impact biocompatibility negatively, since they possess high crystallinity. In this vein, PLLA can be fabricated as a combination of L-lactic acid and D-lactic acid to address this issue. This leads to faster degradation, the lack of crystallinity, and better biocompatibility [187].

Sun et al. created hybrid nanofiber mats using methacrylated gelatin (MeGel) and poly(L-lactic acid) (PLLA) with a nanofibrous structure mimicking the extracellular matrix and hydrogel-like properties, making them potential candidates for advanced wound dressing materials. All crosslinked nanofiber mats exhibited smooth, bead-free fiber morphologies. Those containing MeGel presented significantly improved hydrophilic properties and a higher swelling ratio compared to pure PLLA nanofiber mats. In addition, the UV crosslinking process substantially enhanced the MeGel/PLLA nanofiber mats’ structural stability and mechanical properties. In vitro studies demonstrated excellent biocompatibilities for all crosslinked nanofiber mats, with mats containing MeGel significantly promoting the attachment, growth, and proliferation of human dermal fibroblasts. In conclusion, this study suggests that MeGel/PLLA blend nanofiber mats are promising candidates for wound dressing materials [188].

**Table 2:** Different materials and their properties for use as hydrogels for chronic wound repair.

Classification	Material	Properties	Refs.
Natural polymer hydrogel	Collagen	It presents exceptional biodegradability, low antigenicity, and biocompatibility. It can self-assemble and crosslink, forming robust and stable fibers making it a promising candidate for the development of scaffolds.	[127,128,129]
	Alginate	It presents structural similarities to the extracellular matrix, biocompatibility, lack of toxicity, biodegradability, cost-effectiveness in extraction, and ease of gelation. Due to its poor stability and relatively soft mechanical properties, it is used to combine alginate with other polymers to improve its properties.	[135,138,139,140]
	Hyaluronic acid (HA)	It presents good biocompatibility, biodegradability, bioresorbability, high viscosity, and mechanical stability, leading to an ideal biomaterial for the design and development of non-adhesive, non-thrombogenic, and non-immunogenic scaffolds.	[148,149]
	Chitosan	It presents biodegradability, non-toxicity, non-antigenicity, inertness, bioadhesiveness, and hemostatic effects. It can be modified to create multifunctional constructs that closely resemble the natural matrix.	[151,152,153,154,155,156]
	Gelatin	It presents good biocompatibility, biodegradability, degradation mediated by matrix metalloproteinases, preservation of natural cell adhesion motifs, low antigenicity, and minimal inflammatory response when introduced in vivo due to its degradation process. The functional groups allow for modifications with crosslinkers or therapeutic agents, expanding its versatility as a material applicable to wound healing and tissue regeneration.	[161,162,163,164]
Synthetic polymer hydrogels	Poly(ethylene glycol) (PEG)	It is non-ionic, biocompatible with optimal physicochemical and biological attributes; it typically does not generate an immune response. Chemical modification of PEG scaffolds can further enhance its biological properties.	[173,176]
	Poly(vinyl alcohol) (PVA)	It has reduced protein-binding tendencies, relatively higher elasticity and water content. It has good biocompatibility, non-carcinogenic properties, non-toxicity, swelling characteristics, and bioadhesive features.	[179,180,181]
	Poly(lactic acid) (PLA)	Since lactides lack functionality, they are copolymerized with hydrophilic monomers or conjugated with hydrophilic moieties to create hydrogels. Given that PLA presents high hydrophobicity, modifications to the PLA attributes can be made by copolymerizing or crosslinking with other polymers such as polyethylene oxide, polyethylene glycol, polysaccharides, and polypeptides.	[186]

## 4. Applications of Antioxidant-Incorporated Hydrogels in Chronic Wound Repair

As presented in the previous sections, there are several antioxidants and several materials used as hydrogels. In this section, we are going to highlight different applications of antioxidants incorporated into hydrogels for the healing of chronic wounds (Table 3 shows some of them schematically). In this vein, Ravi and colleagues developed a novel hydrogel system for the targeted delivery of ascorbic acid during wound care, using poly(ethylene glycol) methacrylate to create a graft copolymer (GPMA). The GPMA obtained was covalently and ionically crosslinked, serving as a matrix for ascorbic acid delivery to wounds. An interesting result found by the authors is the change in the swelling pattern due to pH modifications, since a decrease in pH leads to a decrease in the percentage of water uptake by the GMPA matrix. This result means that when the wound starts to heal and the pH decreases, reaching a normal acidic skin pH, it leads to a controlled release of the biomolecule. On the other hand, cytotoxic assays performed on fibroblasts showed that the GMPA material could be used safely for up to 48 h during the wound healing process. The use of GMPA hydrogel leads to sustained released of ascorbic acid, which guarantees the action of the biomolecule over time. In addition, the use of ascorbic acid showed enhancement in wound closure when keratinocytes scratch wound assays were performed. Moreover, as ascorbic acid functions as a cofactor in collagen synthesis, the controlled release of it into the wound microenvironment contributed to the up-regulation of the colα1 gene expression. Taking into account these results, this hydrogel could be used in the wound healing field [189]. 

N-acetylcysteine (NAC) is a frequently used substance because it can hydrolyze to obtain cysteine in the cell, which is a glutathione precursor [190]. In this vein, Qian and colleagues developed an implantable collagen-based scaffold incorporating a mixture of graphene oxide (GO) for the sustained delivery of NAC to assess wound healing in diabetic rats [191]. Diabetic patients often display impaired wound healing associated with altered inflammatory response, poor angiogenesis, and oxidative stress [192]. In this vein, NAC can not only counteract the production of reactive oxygen species but also increase vascularization [190]. On the other hand, graphene oxide has demonstrated the capability to modulate cellular behavior and form crosslinks with biodegradable polymers like collagen to create composite scaffolds [193]. Taking this into account, the authors developed the scaffold, which showed no difference in the physiochemical properties when NAC was added, demonstrating that the addition of this antioxidant did not affect the structure of the scaffold. In addition, the release studies showed that the antioxidant could be released for at least 18 days. Regarding the in vitro studies, it was demonstrated that the scaffolds displayed excellent biocompatibility and exhibited an enhanced capacity to promote collagen secretion by fibroblasts, particularly in a high-glucose environment. Moreover, the authors performed wound healing studies on a 20 mm dorsal full-skin defect in streptozotocin-induced diabetic rats. These results showed a better repair process when the GO-COL-NAC scaffolds were used, since there was uniform and thick collagen bundle deposition. Finally, the authors evaluated the antioxidant stress status, showing that the scaffolds increase the levels of the following antioxidant enzymes: glutathione peroxidase (GPx), catalase (CAT), and superoxide dismutase (SOD), leading to a better antioxidant environment. This scaffold, with its therapeutic potential, could offer valuable advancements in diabetic wound treatment approaches.

Lipoic acid is a potent antioxidant, since it can scavenge free radicals, such as superoxide anion and peroxide radicals [194]. In this context, Zhang and collaborators developed a hyaluronic acid-g-lipoic acid gel for the treatment of diabetic wounds. The aim of this hydrogel was to scavenge reactive oxygen species (ROS) by creating a granular hydrogel made of hyaluronic acid (HA) and acid-g lipoic acid (LA). The characterization of the hydrogel confirmed that LA was successfully grafted with the HA. The synthesis consisted of creating microgels that were assembled by Ag+ through its coordination with disulfide in dithiolane, forming a granular gel. The resulting granular gel exhibits a shear-thinning feature, becoming fluid during extrusion and promptly returning to a solid state afterward. This feature enables easy application across the entire wound area. On the other hand, to evaluate the antioxidant capacity, in vitro assays were performed. The authors incubated endothelial cells with the HA-LA granular hydrogels and found that the gel displayed strong scavenging activity of ROS. Moreover, the antimicrobial activity test showed that the HA-LA granular gel inhibited the growth of *S. aureus* due to the presence of Ag+. Finally, in vivo experiments demonstrate that the HA-LA granular gel effectively reduces excessive ROS at the wound site, enhances the secretion of reparative growth factors, and significantly accelerates both common and diabetic wound healing [195].

Regarding the hydrophobic antioxidants, Shefa and colleagues developed a cellulose nanofiber–polyvinyl alcohol hydrogel loaded with curcumin [196]. Since curcumin has the potential to enhance healing across various stages of the wound healing process, the integration of this antioxidant into a hydrogel system presents an intriguing approach for addressing full-thickness skin wounds. The hydrophobicity of curcumin makes its solubilization a challenge. In this sense, the authors solubilized the antioxidant through a polymer, pluronic F-127, in order to achieve dispersion of the curcumin into the hydrogel, and they developed a crosslinked TEMPO-oxidized cellulose nanofiber–polyvinyl alcohol–curcumin (TOCN-PVA-Cur) hydrogel prepared via a freeze–thaw process, which can release curcumin to contribute to wound healing. Morphology analysis, performed by SEM micrograph, revealed that the inclusion of TOCN increases the porosity of the hydrogel. In order to confirm the incorporation of curcumin in the hydrogel, the authors used the fluorescence property of curcumin using confocal microscopy. On the other hand, the authors performed both in vitro and in vivo studies. The first ones showed that fibroblast cells internalized curcumin within 4 h of incubation, facilitated by the pluronic polymer system. Moreover, the in vivo studies showed that the application of the hydrogels on rats’ full-thickness skin wounds led to an increased percentage of wound closures. These findings underscore the efficacy of delivering curcumin through the developed hydrogel as a promising method to facilitate natural wound healing processes. In addition, the newly developed hydrogel demonstrated efficiency in promoting wound contraction, enhancing collagen organization, and avoiding associated secondary defects and scar formation.

In the same vein, Wang and collaborators also used curcumin as an antioxidant for skin wound healing. The authors developed a composite hydrogel film utilizing silk fibroin (SF) and polyvinyl alcohol (PVA) as scaffolds, and then, they loaded it with curcumin nanoparticles (Cur NPs). As mentioned before, curcumin is a hydrophobic antioxidant. In this sense, the Cur NPs were synthesized through a process involving drop-adding a dichloromethane solution of curcumin into boiling water, assisted by sonication. The combination of the antibacterial nanoparticles of curcumin with the biocompatible SF/PVA hydrogel demonstrated a synergistic effect for wound closure. Antibacterial activity was demonstrated against both Gram-negative (*E. coli*) and Gram-positive (*S. aureus*) bacteria. The biocompatibility of the hydrogel was confirmed, since it led to cell proliferation, confirmed by MTT analysis. On the other hand, wound healing tests were performed using an in vivo model of rats. This test showed that the hydrogels loaded with Cur NPs significantly promoted wound closure and resisted bacterial infection compared to the control. In addition, the authors performed a histological examination of the wound tissue, showing that there were more fibroblasts in the wound sites treated with the hydrogels and accelerated collagen deposition compared to the control. Immunological analysis revealed that the fabricated Cur NP composite hydrogel films not only inhibited inflammation at the wound sites but also promoted angiogenesis during the wound healing process and suppressed the production of inflammatory factors. Moreover, the authors showed that the developed hydrogels presented excellent mechanical properties and good swelling and hydrophilic performance, making them suitable for conforming to various wound shapes. In this sense, the developed composite hydrogel film holds promise as a potential skin wound dressing for the effective treatment of skin wounds [197].

Another interesting antioxidant is the α-tocopherol, also known as vitamin E. In this context, Bergonzi and colleagues designed a biocompatible 3D-printed chitosan scaffold with α-tocopherol, which displayed antioxidant and antimicrobial properties [198]. Regarding the stability assays, the authors found that the manufacturing process and the storage conditions did not reveal significant drug loss. In addition, the chemical and physical characterization demonstrated that the dressings were highly porous, could be dehydrated up to 80%, and could recover more than 90% of water upon 1 h of rehydration. When the stress/strain tests were performed, the elasticity determined was higher than that of human skin, with sufficient resistance to be used in clinical manipulation. The study of the antioxidant activity revealed that the antioxidant action of the scaffolds was α-tocopherol dose-dependent, being almost 80% in less than 1 h. On the other hand, the in vitro studies showed the biocompatibility of the scaffolds with α-tocopherol for over 28 days. Finally, the authors evaluated the antimicrobial activity against Gram-positive (*S. aureus*) and Gram-negative (*P. aeruginosa*) bacteria using the inhibition rings obtained via the Kirby–Bauer technique. These results showed antimicrobial activity against these strains. In this sense, the designed hydrogels, with their combined antioxidant and antimicrobial properties, have potential applications as dressings for the treatment of chronically infected wounds.

**Table 3:** Loading and release strategies, healing mechanisms, and efficacy of antioxidant-loaded hydrogels for skin wound healing.

Hydrogel	Antioxidant	Loading Strategy	Release Method	Healing Mechanism	Efficacy	Refs.
Gellan-g-poly(ethylene glycol) methacrylate matrix (GPMA)	Ascorbic acid (AA)	Diffusion filling method.	Release controlled by swelling behavior and mechanical properties of the hydrogel. GPMA hydrogel showed sustained release of ascorbic acid at pH 5.4 and 7.4. It has pH sensitivity, and the release at lower pH was decreased.	Collagen synthesis, anti-oxidant, and anti-inflammatory activity.	55% wound closure within 24 h (HaCaT cells).	[189]
Graphene oxide-collagen scaffold (GO-COL)	N-acetylcysteine (NAC)	NAC is mixed with the precursor polymer solution, and gelation occurs with the drug within the matrix.	NAC loaded in the scaffolds could be persistently released for at least 18 days, GO-COL hydrogel showed sustained release of NAC.	Resist oxidative stress, promote angiogenesis, accelerate ECM-synthesis, and facilitate epithelization.	58.176% wound closure within 24 h (NIH 3T3 fibroblasts).	[191]
Hyaluronic acid-g-lipoic acid (HA-LA)	Lipoic acid (LA)	Grafted LA forms part of the granular gel, being mixed in the precursor polymer solution.	Controlled release of LA for at least 3 days (dressings changed every 3 days).	Eliminate excessive ROS at the wound site and up-regulate the secretion of reparative growth factor.	Significant promotion of wound healing in both normal and diabetic mice.	[195]
Oxidized cellulose nanofiber-polyvinyl alcohol hydrogel system (TOCN-PVA)	Curcumin (Cur)	Cur solubilization by ethanol (100%) and pluronic^®^ (4%) to be mixed in the precursor solution.	In contact with fluid, Cur is released from the hydrogel system, along with Cur/Plu cargo/misceli. Curcumin release increased dramatically after 1 day of incubation.	Scavenge ROS and lipid peroxidation.	Significant wound contraction after 2 weeks of application in rat skin excisional wound model.	[196]
Silk Fibroin and Polyvinyl Alcohol Composite Hydrogel Film	Curcumin (Cur)	Cur nanoparticles were fabricated and loaded into hydrogel films.	Nanoparticles mediated release of curcumin.	Antibacterial activity, increase in fibroblasts and angiogenesis, inhibit inflammation, and accelerate deposition of collagen.	After 7 days, skin wound remodeling and rebirth were higher on rats’ whole skin injury model.	[197]
Chitosan (3D printed)	α-tocopherol	Dispersion of α-tocopherol in chitosan solution.	Controlled drug release by geometrically complex devices designed for 3D printing.	Radical scavenging activity and antibacterial activity.	High biocompatibility was shown on human fibroblast cultures.	[198]

## 5. Concluding Remarks and Future Directions

The present review discusses the applications of various skin-related antioxidants and their combination with biopolymers to enhance wound healing. Given the fact that chronic wounds affect millions of people worldwide, developing new approaches to treat this condition is clearly a priority. In this vein, the findings presented highlight the significant potential of combining biopolymers with antioxidants in promoting healing. As we have outlined earlier, hydrogels offer noticeable benefits when designing these systems, such as excellent biocompatibility and biodegradability and the ability to be customized, making them attractive candidates for applications in wound care. Thus, an important part of the focus on the advancement of hydrogels for wound healing is the selection of polymers. One of the key contributions of this article is the comprehensive review of various natural biopolymers, including chitosan, alginate, collagen, and hyaluronic acid, among others, and synthetic ones. Each of these biopolymers exhibits unique characteristics that can be tailored to meet specific requirements for antioxidant delivery in wound healing. In parallel, we present a novel approach that merges the use of hydrogels with antioxidants as therapeutic agents to treat wounds. The elucidation of the distinct mechanisms of action of widely used antioxidants provides valuable insights for designing optimized formulations based on the nature and severity of the wound.

To demonstrate the utility of this approach, some of the studies that show the applications of antioxidant-incorporated hydrogels in chronic wound repair are discussed. Within this framework, one of the most important insights is that hydrogels have a positive impact on antioxidant release kinetics. The controlled and sustained release of antioxidants from biopolymers addresses the challenge of maintaining therapeutic levels at the wound site over an extended period. This controlled release not only ensures prolonged antioxidant protection but also minimizes potential side effects associated with rapid and high-dose delivery. However, translating these advancements into practical solutions for improving wound care outcomes is still a challenge. 

As the field of hydrogel-based antioxidant delivery for wound healing progresses, future research should involve multifunctional delivery systems. Indeed, future efforts should focus on hydrogels that not only deliver antioxidants but also possess hemostatic and anti-inflammatory properties. It is worth mentioning that extensive inflammatory infiltration represents a common feature of chronic wounds, which hinders the repair of the wound in a chronological and biological sequence [199]. In parallel, rapid and effective hemostasis is of paramount importance to improve the wound healing process [200]. As a consequence, it may be necessary to incorporate additional agents alongside antioxidants within the hydrogel structure to effectively inhibit inflammation, promote hemostasis, and promote wound healing [201,202].

In conclusion, there is substantial evidence that hydrogel-based systems for antioxidant release represent a promising avenue for advancing the field of skin wound care. However, there is still a considerable amount of work to be done on the formulation strategies, biopolymer and antioxidant selection tailoring specific wound types, and even optimizing release profiles. In addition, the integration of advanced technologies, such as nanotechnology and 3D printing, could further enhance the efficacy and precision of antioxidant delivery systems.

## Data Availability

Not applicable.
